# Antibacterial Effect of Carbosilane Metallodendrimers in Planktonic Cells of Gram-Positive and Gram-Negative Bacteria and *Staphylococcus aureus* Biofilm

**DOI:** 10.3390/biom9090405

**Published:** 2019-08-23

**Authors:** Celia Llamazares, Natalia Sanz del Olmo, Paula Ortega, Rafael Gómez, Juan Soliveri, F. Javier de la Mata, Sandra García-Gallego, José Luis Copa-Patiño

**Affiliations:** 1 Department of Biomedicine and Biotechnology, University of Alcalá, 28805 Madrid, Spain; 2 Department of Organic and Inorganic Chemistry, and Research Institute in Chemistry “Andrés M. del Río” (IQAR), University of Alcalá, 28805 Madrid, Spain; 3 Networking Research Center on Bioengineering, Biomaterials and Nanomedicine (CIBER-BBN), 28029 Madrid, Spain; 4 Institute Ramón y Cajal for Health Research (IRYCIS), 28034 Madrid, Spain

**Keywords:** dendrimer, metallodendrimer, metal, copper, ruthenium, antibacterial, biofilm, *Staphylococcus aureus*, *Escherichia coli*

## Abstract

Antibiotic resistance is currently one of the main threats to public health security. Biofilm formation is a resistance mechanism that is responsible for most human bacterial infections and requires new and effective therapeutic approaches, such as those provided by nanotechnology. In this work, the antibacterial effect of carbosilane metallodendrimers with different metals (copper(II) and ruthenium(II)), ligands (chloride and nitrate) and generations (generation 0, 1 and 2) has been studied using planktonic Gram-positive (*Staphylococcus aureus*) and Gram-negative (*Escherichia coli*) bacteria. Furthermore, the ability of the metallodendrimers to avoid the formation of *S. aureus* biofilms was also evaluated. The results showed a promising biocide activity in both types of planktonic bacteria, especially for first-generation dendrimers, which arises from the metal complexation to the dendrimer. Cu(II) metallodendrimers require lower concentration than Ru(II) counterpart to inhibit the production of *S. aureus* biofilms, but none produce hemolysis at the inhibitory concentrations and can be safely used as antibacterial agents. In particular, the first-generation Cu(II) metallodendrimer with nitrate ligands displayed the most promising properties to continue with further studies in both planktonic cells and biofilms.

## 1. Introduction

*Escherichia coli* and *Staphylococcus aureus* are representatives of Gram-negative and Gram-positive microbes, respectively. The high resistance and ability to produce infections of *S. aureus* demand special attention from health authorities. *S. aureus* is a microbe from the *Staphylococcaceae* family with spherical cells, usually arranged in irregular clusters, similar to a bunch of grapes. Its size ranges from 0.8 to 1.5 microns in diameter, it is immobile and some strains produce an external mucoid capsule that increases its ability to adhere, preventing it from being recognized and reinforcing the antiphagocytic effect that increases the ability to produce infection [1]. In relation to its metabolism, it is facultative anaerobic, coagulase-positive, catalase-positive and oxidase-negative. It grows forming colonies in an optimum temperature range 30–40 °C [2,3], and they can even grow in seawater and ferment glucose, lactose and maltose [1,4,5]. *S. aureus* is an opportunistic pathogen found in the normal human microbiota, at the skin of healthy individuals [6,7]. Between 30 and 50% of healthy adults are colonized, and between 10 and 20% remain persistently colonized [8,9,10]. Although anyone can develop staphylococcal infection, risk-populations include people with chronic conditions or weakened immune system, people who have had surgery and/or those who use a catheter (e.g., dialysis patients) [6,7,11,12,13]. *S. aureus* infections can affect the skin, the bloodstream, bone tissues or the eyes, leading to life-threatening diseases like endocarditis, pneumonia, toxic shock syndrome or keratitis. Most chronic and recurring infections, such as permanent medical device infections [14,15,16,17,18], are related to the production of bacteria biofilm [19,20].

Human bacterial infections are mainly produced by bacteria in a biofilm-mode of growth and not due to planktonically growing bacteria. A biofilm is a sessile community derived from microbes embedded in a matrix of extracellular polymeric substance which exhibit an altered phenotype with respect to growth [6,7,21]. Biofilm formation is divided into four distinct metabolic states: aerobic, fermentative, latent (including persistent very slow-growing cells) and dead cells [22,23]. The antimicrobial resistance of biofilms is explained by the stressed environment, which produces many cells with low metabolic rates, and its ability to act as a diffusion barrier that hinders the penetration of antimicrobial agents. In bacterial infection, biofilm matrix acts as a safe haven, protecting bacterial cells from antibiotics, immune cells and antimicrobial factors [24,25]. Apart from biofilm formation, other types of bacterial resistance can arise from spontaneous mutation or through the genes exchange between different strains or species of bacteria [26]. The evolution of antibiotic resistance is currently one of the main threats to public health security; the first warning came from Alexander Fleming, discoverer of penicillin. Only four years after penicillin introduction in clinic, 14% of *S. aureus* hospital strains were resistant, number that increased to 59% four years later. In the 1980s-90s, resistance exceeded 80% in communities and 95% in most hospitals [27]. 

Unfortunately, biofilm-bacteria are more resistant to conventional antimicrobials and require new approaches, such as those provided by nanotechnology [28,29,30,31]. Metal-based nanoparticles (NPs), carbon-based nanomaterials, as well as polymeric NPs, liposomes and dendrimers have been proposed as biofilm antimicrobials. These NPs not only possess antimicrobial properties of their own, but can also be used as drug delivery systems. Dendrimers are highly branched three-dimensional macromolecules whose structure is globular and monodisperse. These unique properties enabled their evaluation in multiple applications in the field of medicine, including drug carriers to increase bioavailability, gene carriers to protect nucleic acids, specific antitumor systems and broad-spectrum antiviral and antibacterial agents [32]. In the past years, successful dendrimer-based strategies to control microbial contamination and prevent biofilm formation have been proposed [33,34], mainly relying on peptide dendrimers, glycodendrimers, quaternary ammonium dendrimers and metallodendrimers. While exhibiting different modes of action, the antimicrobial activity ultimately is produced by the ability to bind to the negatively charge bacterial cell surface and/or membrane proteins and phospholipids that leads to cell membrane disruption. The presence of metal ions in the nanoparticle can further improve the antimicrobial action through the production of Reactive Oxygen Species (ROS) that induce DNA and mitochondria damage, cell membrane disruption and interruption of transmembrane electron transport [28]. Most antibacterial metallodendrimers reported in the literature rely on the presence of Ag(I) or Ag(0), well-known antimicrobial metal [35], and few examples include other metals such as Cu(II) and Zn(II) [36].

Although the functional groups in the dendrimer surface determine most of the properties of the nanoparticle, the nature of the scaffold also influences its biological activity. As an example, the hydrophobic, stable and flexible scaffold in carbosilane dendrimers enhances their interaction with biological membranes and enables a potent biological activity even with low generation dendrimers [37]. Our group has recently described the use of Schiff-base carbosilane dendrimers as promising delivery agents of Ru(II) and Cu(II) metallodrugs in cancer therapy [37,38,39]. These metallodendrimers produced a significant tumor size reduction in an *in vivo* mice model of resistant prostate cancer, with no signs of toxicity during the experiment.

Herein, we present carbosilane metallodendrimers as an alternative to traditional antibiotics and evaluate the influence of different parameters—dendrimer generation, metal ion, ligand—in the biocide effect against planktonic cells and biofilms of *S. aureus*. Promising bacteriostatic and bactericide activity and no hemolysis were found, especially for first-generation dendrimers.

## 2. Materials and Methods

### 2.1. Metal Complexes and Metallodendrimers

**Figure 1:**
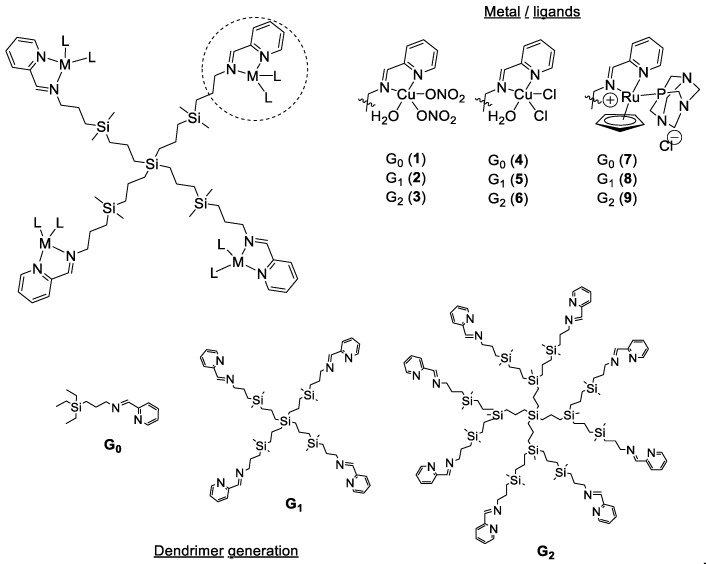
Chemical representation of the tested Schiff-base carbosilane metallodendrimers, highlighting the structural parameters studied.

The selected metal complexes comprised two families of copper (II) carbosilane complexes, with nitrate (**1**–**3**) and chloride (**4**–**6**) ligands, and a family of ruthenium (II) carbosilane complexes (**7**–**9**) (Figure 1). These complexes were selected in order to study the influence of the metal, the ligand and the generation on their antibacterial activity. The Cu(II) and Ru(II) mononuclear complexes and first and second-generation metallodendrimers were synthesized according to previously published protocols [37,38,39]. The different metal salts used as negative control included Cu(NO_3_)_2_·H_2_O, CuCl_2_·2H_2_O and RuCl_3_·H_2_O.

Serial dilutions of the different biocides were prepared in sterile distilled water for copper complexes with nitrate ligands **1**–**3** and ruthenium complexes **7**–**9** due to their good solubility, and in dimethyl sulfoxide (DMSO) : water (1:99 at the highest concentration) for chloride copper complexes **4**–**6**. The effect of DMSO at the different concentrations was evaluated in an independent study, ruling out any possible toxicity for antibacterial assays.

### 2.2. Bacterial Strains

The microorganisms used in the assays were a strain of *Escherichia coli* (CECT 515, Gram-negative) and/or a strain of *Staphylococcus aureus* (CECT 240, Gram-positive) provided by the Spanish Type Culture Collection (CECT) in lyophilized form.

### 2.3. Zeta Potential Evaluation

Zeta potential was measured using a Photon Correlation spectrometer Zetasizer Nano ZS, Malvern Instruments (UK). Helmholtz-Smoluchowski’s equation was used to calculate the final value. Five measurements in seven cycles of each sample were made. Compounds were measured in distilled water at a concentration of 30 µM. The data were analyzed using Zetasizer Software (version 7.11, Malvern Instruments Ltd., Malvern, UK).

### 2.4. In Vitro Antibacterial Activity Tests against Planktonic Cells

The assay was based on the ISO 20776-1:2006 protocol. After inoculation, the microorganism was incubated with biocides and controls in sterile 96-well plates, at each of the 13 concentrations. All samples were evaluated in triplicate. The negative controls comprise the inoculum—sample without biocide—to test the correct growth of the microorganism; the biocide—sample without inoculum; and the culture medium, sample without inoculum and biocide. Negative controls were used to rule out any contamination or any additional effects which could affect the correct reading of the plate. The plates were incubated for 24 h at 37 °C. Afterwards, the plates were analyzed using an Ultra Microplate reader (BIO-TEK Instruments, model ELx808, Winooski, Vermont, United States), using a wavelength of 630 nm. The results were collected to obtain the Minimum Inhibitory Concentration (MIC) of the biocide. Subsequently, 5 µL of one of the repetitions of each biocide concentration and of the controls were deposited on a petri dish containing solid medium. This test was performed in duplicate and incubated for 24 h to obtain the Minimum Bactericidal Concentration (MBC) values. For the tests of antibacterial activity using 96-well plates, medium Muller-Hinton (Scharlau, Madrid, Spain, ref. 02–136) was used as culture medium. For the growth of bacteria in petri dish, Plate Account Agar (PCA) (Scharlau, ref. 01–161) was used as culture medium.

### 2.5. In Vitro Antibacterial Activity Tests to Prevent S. aureus Biofilm Formation

The assay was based on the ISO 20776–1:2006 protocol. Bacteria were cultured in PCA petri dish at 37 °C for 24 h and then some colonies were taken and added to a tube containing Bacto Tryptic Soy Broth (Becton, Dickinson and Company, Franklin Lakes, NJ, United States, ref. 211825) until 0.5 units of McFarland scale was obtained. The tube was incubated at 37 °C for 20 h. Afterwards, a dilution of 1:100 was made with the same medium (inoculum solution). An aliquot of 200 µL of inoculum solution was mixed with 50 µL of each of the 16 concentrations of the biocides and the controls in sterile 96-well plates and incubated for 10 h at 28 °C. The different concentrations of biocide were evaluated by triplicate and controls of inoculum, biocide and culture medium were tested as well. Biofilm formation was measured as follows: first, the total absorbance of each well was measured using an Ultra Microplate reader (BIO-TEK Instruments, model ELx808). After that, the supernatant (planktonic cells) was removed and added to new 96-well plates and the absorbance was measured again, to determine the Minimum Inhibitory Concentration (MIC). In the first 96-well plate, remaining biofilms were stained with 1% violet crystal in water for 15 min. After removing excess dye with PSB (phosphate buffered saline, 10 mM, three gentle washing cycles), the plate was dried and 200 µL of acetic acid (33% water solution) was added to remove the dye inside the cells. The acetic acid solution was extracted from the well and deposited in another new 96-well plate in order to measure the absorbance of each well and determine the Minimum Biofilm Inhibitory Concentration (MBIC). In all cases, a 630 nm wavelength was used. The Minimum Bactericidal Concentration for Biofilms (MBC-B) was obtained using 5 µL of one of the replicates of each biocide concentration and controls for inoculating a petri dish with PCA medium. The plate was incubated for 24 h at 37 °C, and the assay was performed in duplicate.

### 2.6. Hemolysis Evaluation

The assay was adapted from the ISO 10993–4 protocol. Erythrocytes were isolated from lamb blood (RBC, Oxoid sheep erythrocytes) by centrifugation at 800× *g* for 10 min, washed three times with PBS 10 mM, pH 7.4 and finally resuspended to a final volume of 2 mL of PBS 10 mM. A 1:50 dilution in PBS 10 mM was used to analyze the hemolytic effect of the metallodendrimers. A 20 µL aliquot of the dendrimers in decreasing final concentrations from 32 to 0.25 mg/L was added to 180 µL of the erythrocytes solution. Then, the samples were incubated at 37 °C for 2 h. Absorbance was measured at 540 nm using BioTek Epoch 2 spectrophotometer. Equation (1):

(1) $$H\left(\text{\%}\right)=\frac{\left[Abs\left(dendrimer\right)-Abs\left(negative\;control\right)\right]\times 70}{Abs\left(positive\;control\right)}$$

The percentage of hemolysis was calculated using Equation (1). The negative control was PBS 10 mM (20 µL + 180 µL of erythrocyte solution) and the positive control was Triton X-100 1% (20 µL + 180 µL of erythrocyte solution). The latter control is considered to produce 70% hemolysis. The term “Abs(dendrimer)” is calculated by subtracting the absorbance of the compound alone to the absorbance of the compound-treated erythrocytes.

## 3. Results

### 3.1. Surface Charge of Metal Complexes and Metallodendrimers

Zeta potential measurements provide information about the surface charge of the tested compounds. The metal complexes and metallodendrimers selected for this study exhibited cationic properties, according to the Z-potential measurements (Table 1). As expected, the surface charge on the dendrimers increased from G0 to G2 according to the increase in the number of branches and the number of positive charges in the periphery. Furthermore, nitrate-containing complexes **1**–**3** presented a higher positive charge than chloride-containing systems **4**–**6**, with values in the range 14.79–39.23 and 10.45–37.48 mV, respectively [40]. This behavior is ascribed to the more labile properties of the nitrate ligands in solution that easily expose the copper charge. Regarding ruthenium complexes **7**–**9**, the higher Z-potential values in the range 18.70–44.90 mV indicated an even higher overall cationic charge in the molecule. 

### 3.2. Antibacterial Activity of Carbosilane Metallodendrimers on Planktonic Cells

The antibacterial behavior of Cu(II) and Ru(II) metallodendrimers **1**–**9** was evaluated towards two different families of bacteria: *Staphylococcus aureus* as a model of Gram-positive bacteria, and *Escherichia coli* as a model of Gram-negative bacteria. Table 1 summarizes the obtained values for the Minimum Inhibitory Concentration (MIC) and the Minimum Bactericidal Concentration (MBC) for each of the synthesized complexes, as well as the metallic salts used as precursors.

The data shown in Table 1 indicates that carbosilane metallodendrimers are promising antibacterial agents towards Gram-positive and Gram-negative bacteria. The different metal salts used as control—Cu(NO_3_)_2_, CuCl_2_ and RuCl_3_—showed no antibacterial effect. However, mononuclear complexes **1**, **4** and **7** exhibited certain activity, especially higher towards *S. aureus*. These results confirm that the antibacterial activity is ascribed to the metal complexation to the ligand. The metallodendrimers, mainly first-generation systems **2**, **5** and **8**, with 4 metal atoms in their periphery, are potent agents towards both types of bacteria, displaying MIC values in the range 2–8 mg/L for *S. aureus* and 4–16 mg/L for *E. coli*. Second-generation counterparts revealed lower activity, despite the increase in the amount of metal atoms in their structure. Overall, we can conclude that in planktonic cells the antibacterial activity follows the trend Ru(II) complexes > Cu(II) chloride complexes > Cu(II) nitrate complexes, with the first-generation dendrimers as the more potent members in each family. In fact, the antibacterial effect among first-generation systems is comparable.

### 3.3. Antibacterial Activity of Carbosilane Metallodendrimers on Preventing S. aureus Biofilm Formation

**Figure 2:**
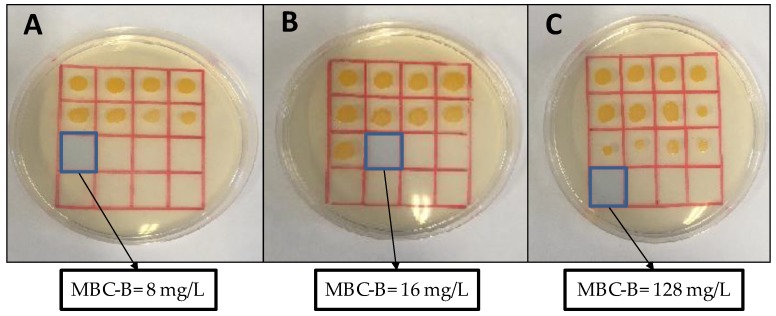
Effect of first-generation metallodendrimers in preventing the formation of *S. aureus* biofilms. (**A**) Compound **2**, with Cu(II) nitrate complex; (**B**) compound **5**, with Cu(II) chloride complex; and (**C**) compound **8**, with Ru(II) Cp/PTA complex. The Minimum Bactericidal Concentration for Biofilms (MBC-B) represents the minimal concentration of the metallodendrimer that can kill the microorganism in the conditions used to produce a biofilm.

Considering the involvement of *S. aureus* biofilms in numerous human infections, we selected the potent first-generation copper metallodendrimers **2** and **5** and the ruthenium counterpart **8** and evaluated their capacity to prevent the formation of *S. aureus* biofilms. The results are summarized in Table 2 and Figure 2. 

The MIC represents the minimal concentration that inhibits the growth of the microorganisms while the MBIC indicates the minimal concentration that inhibits the formation of the biofilm although not the growth of the microorganisms. Therefore, MBIC value is equal (e.g., compounds **5** and **8**) or lower (e.g., compound **2**) than MIC value. The MBC-B indicates the minimal concentration that kills the microorganisms and it is equal (e.g., compound **2**) or higher (e.g., compounds **5** and **8**) than MIC value.

The results indicate that carbosilane metallodendrimers are also effective inhibitors of the formation of *S. aureus* biofilms. Copper metallodendrimer **2**, with nitrate ligands, kept the potent bacteriostatic and bactericide effect observed in planktonic cells (MIC(planktonic) = MBIC = 4 mg/L and MBC(planktonic) = MBC-B = 8 mg/L). The ligand exchange, from nitrate to chloride, produced a decrease in the bacteriostatic and bactericide effect when *S. aureus* are prompt to form biofilms (MBIC = 8 mg/L and MBC-B = 16 mg/L), and the same happened for the metal exchange, from copper to ruthenium (MBIC = 32 mg/L and MBC-B = 128 mg/L). It is not surprising that the value of MIC in the conditions of biofilm production are higher than in the case of planktonic cells, because the concentration of microorganisms is higher in the former case. Overall, the best activity was found with compound **2** because MIC and MBC values are coincident (8 mg/L). In the case of compounds **5** and **8**, the MBC value is higher than the MIC value, twice and four times, respectively, thus increasing the potential toxicity of the metallodendrimer. 

### 3.4. Hemolysis

**Figure 3:**
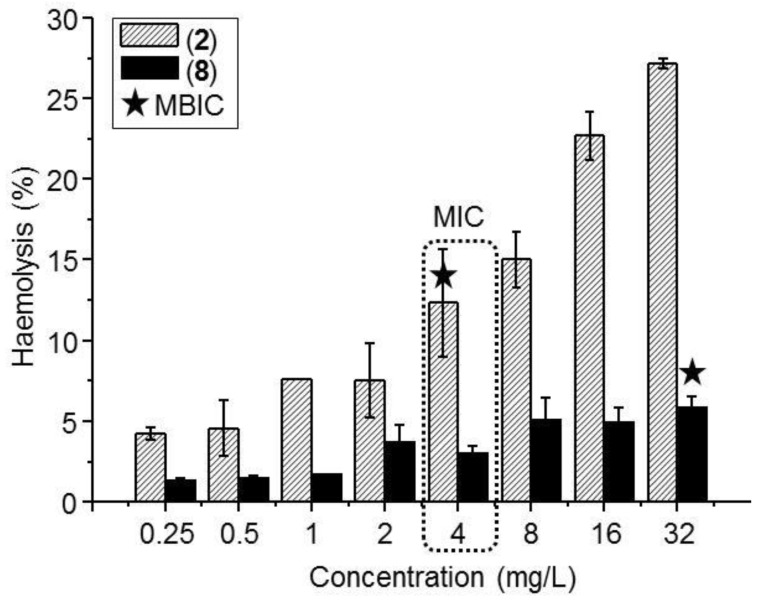
Erythrocyte hemolysis induced by selected carbosilane metallodendrimers **2** (copper nitrate complex) and **8** (ruthenium Cp/PTA complex) after 2 h incubation. The concentration ranged 0.25-32 mg/L. The Minimum Inhibitory Concentration (MIC) and the Minimum Biofilm Inhibitory Concentration (MBIC) for both compounds are highlighted. Results are mean ± S.E.M (standard error of the mean).

Positively-charged molecules frequently exhibit toxicity through the interaction with cells membrane and subsequent destruction. The interaction with erythrocyte membranes produces the release of hemoglobin, among other components, and can be used to measure the toxicity of a new drug. The hemolysis assay is a common test used as a first screening to rule out any toxicity for the therapeutic use of a compound [41]. The hemolysis produced by selected metallodendrimers after 2 h incubation is depicted in Figure 3. 

The assay results showed an increase in hemolytic behavior when increasing dendrimer concentration. At all concentrations tested, ruthenium system **8** produces a substantially lower hemolysis, i.e., at the higher concentration tested it exhibited 5% hemolysis compared to the 27% produced by the copper counterpart **2**. Chloride-containing copper metallodendrimer **5** has been reported to exhibit higher hemolytic behavior than the nitrate analogue [40]. In any case, the metallodendrimers produce low hemolysis at the MIC value in planktonic cells (4 mg/L) and in biofilm-forming cells (4 mg/L for **2**, 32 mg/L for **8**).

## 4. Discussion

Schiff-base carbosilane dendrimers are promising carriers of metallodrugs. Our previous studies confirmed that the resultant metallodendrimers, containing Cu(II) and Ru(II) complexes, displayed promising antitumoral activity in both *in vitro* and *in vivo* assays [37,38,39]. Furthermore, the different structural parameters—dendrimer generation, metal ion, ligands—influenced the biological activity of the final metallodendrimers. Herein, we discuss the bacteriostatic and bactericide properties of selected carbosilane metallodendrimers and the influence of these structural parameters on the antimicrobial activity (Figure 1).

The metal complexes and metallodendrimers selected for this study exhibit cationic properties, according to the Z-potential measurements (Table 1) [40]. Therefore, they can interact with the negatively charged bacterial membranes through electrostatic interactions and disrupt the membrane. This mechanism has been previously described for other cationic dendrimers [33].

The antibacterial effect of carbosilane metallodendrimers was evaluated by measuring the bacteriostatic (MIC) and bactericide (MBC) properties towards Gram-positive and Gram-negative bacteria (Table 1). From this assay, several conclusions were drawn: 1) the antibacterial effect arises from the metal complexation to the ligand, according to the inactivity of the metal salts used as control. Furthermore, previous studies have demonstrated that the Cu(II) and Ru(II) complexes herein reported are completely stable and no metal release has been observed due to the chelating effect of the iminopyridine ligands. Conversely, most antibacterial metallodendrimers reported in the literature rely on the complexation of silver or other metals and the subsequent release of metal ions to disrupt the protein structure [33]. 2) In first-generation dendrimers the dendritic effect becomes evident, with potent antibacterial activity. Surprisingly, mononuclear and second-generation complexes showed similar antibacterial activities and ruled out a direct relationship between antibacterial activity and number of metal atoms. This unique behavior of first-generation carbosilane metallodendrimers has been previously reported [37,38,39] and differs from most of other types dendritic scaffolds. As an example, polyamide Pt(II) and Pd(II) metallodendrimers display an increasing antibacterial effect when increasing generation, being second-generation dendrimer with 12 active groups the most effective with MIC values of 70 μg/mL (PdG2) and 78 μg/mL (PtG2) against *E. coli* [42]. Conversely, four active groups in the first-generation carbosilane dendrimer **2** are enough to reach MIC values of 4 mg/L towards *E. coli*. This is translated into a resources saving—reagents, time, money—when preparing the antibacterial agent. 3) Carbosilane metallodendrimers are potential broad-spectrum antibiotics, with potent activity in both *S. aureus* (Gram-positive) and *E. coli* (Gram-negative) bacteria. Other dendrimers, such as poly(amidoamine) (PAMAM) on titanium substrates described by Wang et al. [43] inhibited Gram-negative bacteria and to less extent Gram-positive *S. aureus*. The authors indicated that the negatively charged lipopolysaccharide in Gram-negative bacteria facilitates dendrimer binding to the membrane and subsequent disruption, while dendrimers barely disrupt the crosslinked peptidoglycan in Gram-positive bacteria. 4) The cationic charge increases with generation, unlike the antibacterial properties. It becomes evident that the antibacterial properties are not directly related to the cationic charge at the dendrimer surface and other mechanisms may be involved related to the presence of the metal ions. Silver and other metal nanoparticles (e.g., ZnO, CuO, SiO_2_) show good antimicrobial effects on drug-resistant strains as well as prevention of biofilm formation and eradication. They have been reported to use antimicrobial mechanisms involving (1) toxic metal ion release, (2) bacterial membrane disruption and (3) reactive oxygen species production [28]. 5) As shown in Table 1, the overall antibacterial activity in planktonic cells follows the trend Ru(II) complexes > Cu(II) chloride complexes > Cu(II) nitrate complexes, with the first-generation dendrimers being the more potent members in each family and exhibiting comparable activity.

The potent antibacterial effect of first-generation dendrimers was subsequently tested for preventing the formation of *S. aureus* biofilms (Table 2, Figure 2). Most human infections produced by *S. aureus* are due to its biofilm-mode of growth, which is highly resistant to traditional antibiotics. In this case, Cu(II) metallodendrimers still keep the potent activity, with subtle differences between nitrate and chloride complexes that point to a higher activity in nitrate systems. Indeed, metallodendrimer **2** exhibited the lowest and coincident MIC and MBC values (8 mg/L) for biofilm formation, thus decreasing the potential toxicity of the antibacterial agent. A similar effect towards cancer cells has been previously reported [37]. Using Electron Paramagnetic Resonance (EPR) analysis, we confirmed that the change of the Cu(II) counter-ion—from nitrate to chloride—produced an increased relative amount and strength of interaction of the dendrimer with model membranes. Interestingly, the stabilization effect observed in chloride dendrimers produced a lower toxicity towards cancer cells. Furthermore, in water solution, the NO_3_^−^ groups are more labile than the Cl^−^ ligands and are easily released, increasing the overall positive charge in the metallodendrimer. The higher cationic charge and the lower membrane stabilization may explain the more potent biocide activity of nitrate metallodendrimer **2** in the present experiments. The ruthenium counterpart **8** showed a potent activity in planktonic cells, lower for biofilm mode-of-growth planktonic cells. The significant morphological and physiological differences between planktonic cells in “normal” and biofilm-forming modes of growth could be responsible of the different behavior of our metallodendrimers. 

Finally, and considering the cationic nature of the tested metallodendrimers, we evaluated the hemolysis of first-generation derivatives (Figure 3). Carbosilane metallodendrimers did not produce hemolysis at the MIC concentrations and can be safely used as antibacterial agents. The hemolytic trend was **8** < **2** < **5**, confirming the influence of both the metal ion and the ligands on the interaction with erythrocytes membrane. Despite the similar Z-potential between copper complex **2** and ruthenium counterpart **5**, especially low hemolysis was observed for the Ru(II) derivative which can be ascribed to macromolecules aggregation that decrease the number of available charges. These results had been also observed in a longer exposure (24 h) using erythrocytes from healthy human donors, obtaining around 5% hemolysis at these concentrations [40].

## 5. Conclusions

Nanotechnology opens new avenues in the treatment of resistant bacteria infections. In particular, the unique properties of dendrimers—monodispersity and multivalency—enable the accurate design of effective treatments by establishing an exact structure-to-activity relationship. A wise selection of dendritic scaffold, generation, metal complex and ligand can lead to potent broad-spectrum antibiotics that can overcome the current limitations of traditional therapies. For example, herein we found that metallodendrimer **2** is the most promising system among those tested. It is stable, water-soluble and exhibits potent bacteriostatic and bactericide effect in planktonic *S. aureus* and *E. coli*. Furthermore, it prevents the formation of *S. aureus* biofilms at a low concentration. Importantly, at the working concentrations, it is not hemotoxic. Further studies to gain insight into the mechanism of action and the *in vivo* activity are currently under way.

## Figures and Tables

**Table 1:** Bacteriostatic (Minimum Inhibitory Concentration, MIC) and bactericide (Minimum Bactericidal Concentration, MBC) effect of carbosilane metallodendrimers in planktonic cells and comparative values of Z-potential.

Compound	Metal Atoms	Zeta Potential, [mV]	*S. aureus*		*E. coli*	
			MIC [mg/L]	MBC [mg/L]	MIC [mg/L]	MBC [mg/L]
Cu(NO_3_)_2_	1	-	>512	>512	>512	>512
**1**	1	14.79 ± 1.92 ^a^	128	128	128	256
**2**	4	25.90 ± 2.32 ^a^	4	8	4	4
**3**	8	39.23 ± 3.78 ^a^	256	256	256	256
CuCl_2_	1	-	>512	>512	>512	>512
**4**	1	10.45 ± 1.25 ^a^	64	64	256	256
**5**	4	19.68 ± 1.78 ^a^	2	4	8	8
**6**	8	37.48 ± 3.09 ^a^	32	32	64	64
RuCl_3_	1	-	>512	>512	>512	>512
**7**	1	18.70 ± 4.21	16	16	64	64
**8**	4	25.62 ± 4.70	4	4	16	16
**9**	8	44.90 ± 5.08	16	32	128	128

^a^ previously published results [40].

**Table 2:** Bacteriostatic effect (Minimum Inhibitory Concentration, MIC, and Minimum Biofilm Inhibitory Concentration, MBIC) of first-generation metallodendrimers in preventing *S. aureus* biofilm formation.

Compound	Metal Atoms	*S. aureus* Biofilms	
		MIC [mg/L]	MBIC [mg/L]
**2**	4	8	4
**5**	4	8	8
**8**	4	32	32
